# Supplementary material for: Comparative Transcriptome Analysis of Two Sweet Sorghum Genotypes with Different Salt Tolerance Abilities to Reveal the Mechanism of Salt Tolerance
Source: Int J Mol Sci. 2022 Feb 18;23(4):2272. doi: 10.3390/ijms23042272 (PMC8877675; doi:10.3390/ijms23042272)
Supplement: Supplementary file 1 [file ijms-23-02272-s001.zip › ijms-1568163-supplymentary.pdf]

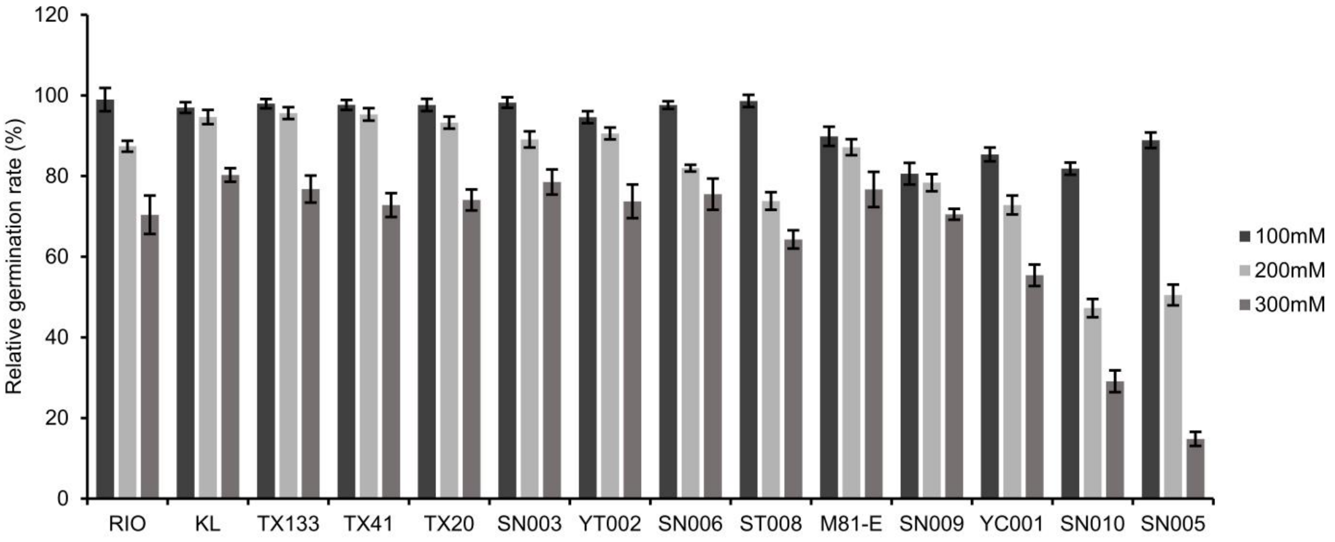

Figure S1. The relative germination rate of 14 sweet sorghum genotypes at 7 days under 100 mM, 200 mM and 300 mM NaCl concentrations. The germination rates of SN005, SN010 and YC001 were significantly different from that of Rio at different salt concentrations (100 mM, 200 mM and 300 mM). The germination rates of SN009 and M81-E were significantly different from that of Rio at 100 mM salt treatment.

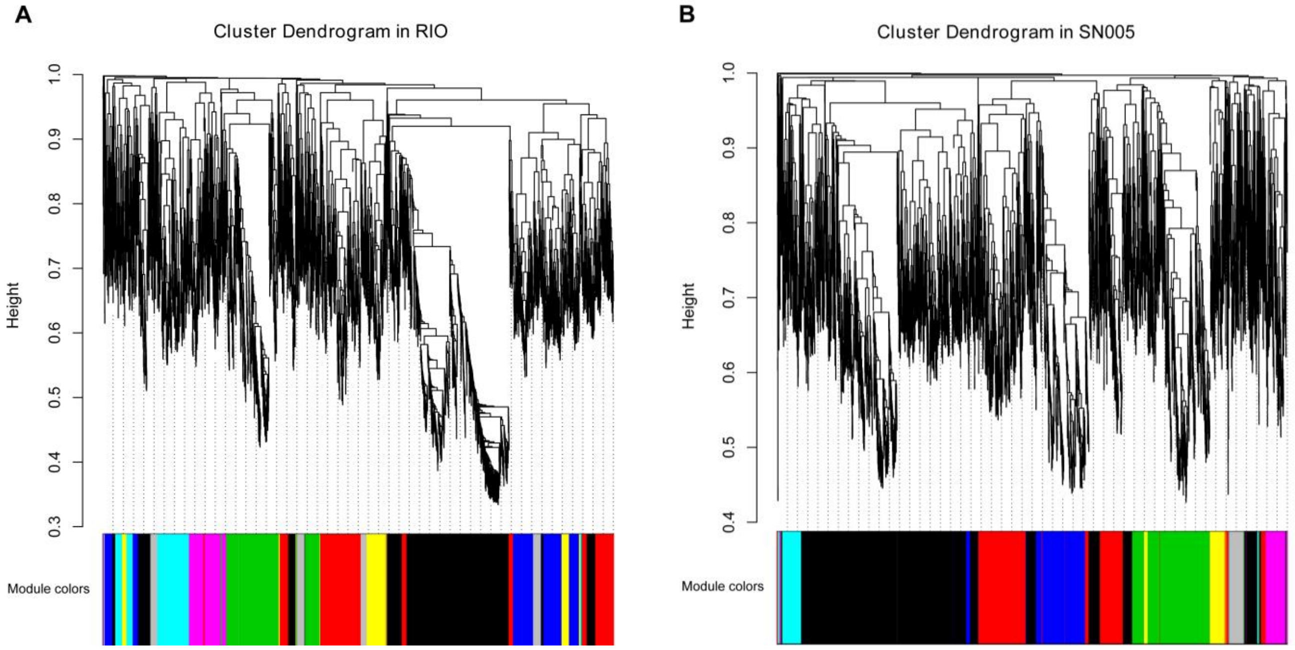

Figure S2. Hierarchical cluster tree and co-expression modules of DEGs identified by weighted correlation network analysis (WGCNA). Each leaf in cluster tree is one gene and different co-expression modules labeled by different colors.

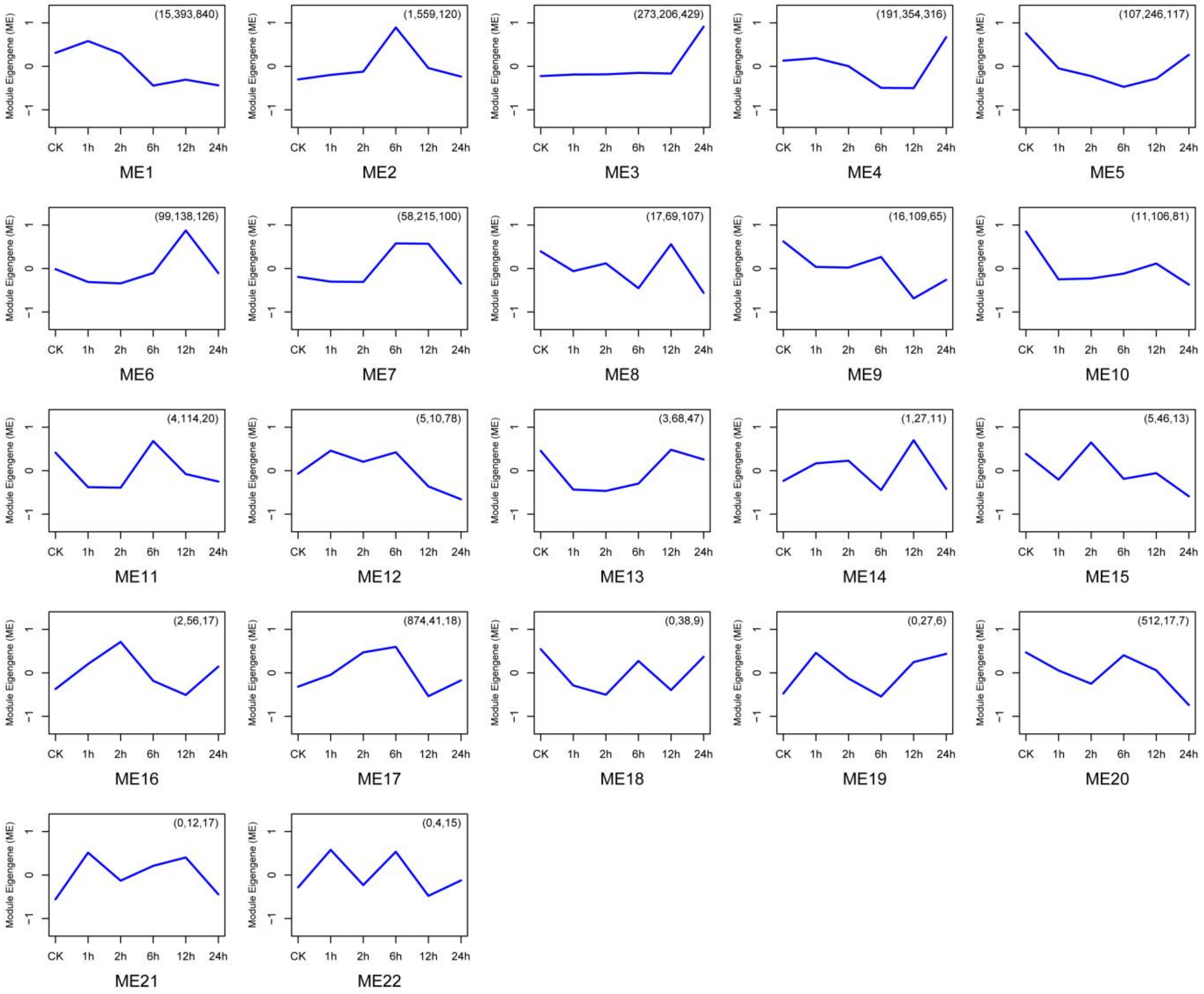

Figure S3. Gene expression pattern of co-expression modules identified by weighted correlation network analysis (WGCNA). Gene expression patterns were presented by the dynamics of module eigengene (ME) values. The numbers in the upper right corner represented the gene number of common response gene set, RIO specific response gene set and SN005 specific response gene set, respectively.
